# Cumulative effects of exercise training and consumption of propolis on managing diabetic dyslipidemia in adult women: a single-blind, randomized, controlled trial with pre–post-intervention assessments

**DOI:** 10.1186/s12576-023-00872-6

**Published:** 2023-08-05

**Authors:** Fatemeh Moayedi, Farzaneh Taghian, Khosro Jalali Dehkordi, Seyed Ali Hosseini

**Affiliations:** 1 Department of Sports Physiology, School of Sports Sciences, Isfahan (Khorasgan) Branch, Islamic Azad University, Isfahan, Iran; 2 Department of Sports Physiology, Marvdasht Branch, Islamic Azad University, Marvdasht, Iran

**Keywords:** Exercise training, Propolis, C1QTNF12, SFRP5, Computational molecular biology

## Abstract

Dyslipidemia is an imbalance of various lipids, and propolis, as a natural resinous viscos mixture made by *Apis mellifera* L. could improve in this condition. In this single-blind, randomized trial, 60 women with type 2 diabetes and dyslipidemia were divided into four groups: (1) the patients who did not apply the combined training and 500 mg propolis capsules supplement (Control group); (2) subjects performed combined training, including aerobic and resistance training (EXR); (3) subjects received the 500 mg propolis supplement capsules (SUPP); (4) Subjects performed combined training along with receiving the 500 mg propolis supplement capsules (EXR + SUPP). We evaluated the concentration of CTRP12, SFRP5, interleukin-6 (IL6), superoxide dismutase (SOD), malondialdehyde (MDA), adiponectin, and total antioxidant capacity (TAC) before and after the intervention. MDA, TAC, IL6, CTRP12, SFRP5 IL6, adiponectin, and lipid profile levels ameliorated in the EXR + SUPP group. We found that 8 weeks of treatment by combined exercise training and propolis supplement decreased inflammation activity and increased antioxidant defense in women with diabetic dyslipidemia.

*Trial registration* This study was registered in the Iranian Registry of Clinical Trials; IRCT code: IRCT20211229053561N1

## Introduction

Dyslipidemia is an imbalance of lipids, especially cholesterol, high-density lipoprotein cholesterol (HDL-C), low-density lipoprotein cholesterol (LDL-C), and triglycerides [1, 2]. The significant rationales of dyslipidemia are induced by inappropriate nutrition, smoking, or a hereditary predisposition [2]. Based on the literature review, dyslipidemia can progress to many serious metabolic problems such as obesity, heart disease, hypertension, fatty liver, chronic inflammation, oxidative stress, and Type 2 diabetes (T2D) [3–7]. In order to effectively manage the growing epidemic of T2D and its associated health issues, it has been designated as one of the four primary non-communicable diseases (NCDs) requiring prompt medical treatment [8]. A rising body of research suggested that T2D is a serious metabolic illness affecting over 400 million people worldwide, and estimated that this figure may exceed 600 million by 2040 [9, 10]. Moreover, the epidemiological study estimated that the prevalence of diabetes and diabetes complications in Iran will reach 9.2 million individuals by 2030 [11–13]. According to epidemiological evidence, prevalence of type 2 diabetes (T2D) and dyslipidemia have been estimated at approximately 30% of the Iranian population [14, 15]. Although the mechanisms of T2D and dyslipidemia are understood to a great extent, comprehensive data on protein functions, protein–protein interactions (PPIs) networks, molecular signaling pathways, and influential pathomechanisms could lead to comprehending the pathogenesis of diabetes with dyslipidemia status, molecular biological etiology, managing, prevention, and treatment of diabesity status [16]. Numerous endocrine variables and hormones are implicated in inter-tissue communication and maintaining the body's energy balance [1]. Evidence indicated that adipokines such as Adipolin/C1qdc2/CTRP12 and secreted frizzled-related protein 5 (SFRP5) could regulate and manage metabolic processes, including insulin sensitivity, glucose uptake, fatty acids metabolism, and inflammation [17–19]. SFRP5 has been shown to exert anti-inflammatory activity via antagonizing the non-canonical wingless-type family member 5A (WNT5A) signaling pathways [20, 21]. Type 2 diabetes, dyslipidemia, obesity, and atherosclerosis are all inflammation-related diseases, with the WNT5A protein as a critical signaling molecule in the pathogenesis of various inflammatory disorders [21–25]. Hence, SFRP5 is a crucial protein at the intersection between obesity, dyslipidemia, and T2DM.

On the other hand, Adipolin, assigned with the alias names CTRP12, FAM132A, and C1QTNF12, is a novel adipokine with anti-inflammatory and insulin-sensitizing properties that are predominantly expressed in adipose tissue [18]. Disruption of these adipocytokines' regulatory mechanisms results in metabolic disorders such as inflammation, dyslipidemias, and T2D. Ample evidence demonstrated that Adipolin/C1qdc2/CTRP12 and SFRP5 could regulate and improve glucose metabolism, inflammation level, insulin resistance (IR), and dyslipidemia. Moreover, based on the evidence, the up-regulation of the CTRP12 in obese and ob/ob fully increased insulin sensitivity and glucose uptake. These studies revealed that CTRP12 might be a potential approach for improving and management of T2D and dyslipidemias condition [17]. Multiple studies indicated that a pro-inflammatory state is a component of T2D with dyslipidemia and a correlation between high Interleukin 6 (IL-6) levels and subjects with type 2 diabetes and dyslipidemia risk factors has been observed [26]. There is mounting evidence that ncRNAs play an essential role in lipid (fat) metabolism, namely in the breakdown and recycling of triglycerides, cholesterol, and lipoproteins. Additionally, ncRNAs may control hepatic fatty acid accumulation by up- or down-regulating downstream molecules involved in fatty acid metabolism [27–29].

The common treatment options for diabetes in Iran include calorie restrictions, diet control, lifestyle management, regular exercise training, oral glucose-lowering medications, monotherapy with insulin administration, and combination therapy with insulin [12]. Complementary medicine is a prospect incorporated with conventional medicine, although it is not considered a replacement for it. Conventional medical therapy is substituted by alternative medicine. Most complementary and alternative medicine forms have been studied less thoroughly than conventional medicine. Consumption of dietary supplements, which may include vitamins, minerals, herbs, and botanicals, is one kind of successful supplemental therapy [30]. Complementary medicine systems have suggested regular exercise and physical activity as therapeutic approaches. Besides natural products, regular exercise training in the long term can substantially impact the body's performance, metabolism, and hormone system activity and ameliorate disorders' hallmarks with limited side effects [1]. Abedpoor et al., in the review study, indicated that regular physical activity with moderate–high intensity (65–90 Vo_2max_) created cross-talk between adipose tissue and other tissues and, through management of adipose tissue function, could be improved pathophysiological hallmarks [1]. A significant body of literature supports that sedentary lifestyles contribute to chronic diseases such as T2D, obesity, fatty liver, neurological disorders, cardiovascular disease, and inflammatory bowel disease. Physical exercise can reduce metabolic diseases by stimulating metabolism, mitochondrial biogenesis, thermogenesis, glucose absorption, and β-oxidation [1, 31, 32]. Furthermore, Akbarian and colleagues revealed that a high-fat diet (HFD) and advanced glycation end-products diet (AGEs) containing 45% HFD, 60% HFD, and 45% AGEs-HFD, or 60% AGEs-HFD induces diabetes and obesity in the C57BL/6 male mice [33]. The HF diet could result in an imbalance in lipids index and trigger metabolic disorders/syndrome.

Propolis, a natural resinous viscos mixture is made by *Apis mellifera* L. Immense evidence has demonstrated that propolis compounds could ameliorate type 2 diabetes with dyslipidemia. naringenin, cinnamic acid, chalcone, kaempferol, chrysin, galangin, tectochrysin, and sakuranetin have antioxidative activity and anti-inflammatory effects. Ample evidence supported this hypothesis that propolis' effective constituents could benefit glucose metabolism, hyperglycemia, lipid profile, dyslipidemia, and inflammation [34, 35].

In this single-blind, randomized, and controlled trial, we evaluated the role of the combined exercise training (aerobic and resistance training) along with consumption of the 500 mg propolis on the anti-inflammation activity, antioxidant, and oxidant status in diabetic dyslipidemia conditions in adult women. Interestingly, we analyzed the closest microarray datasets obtained from the GEO database to determine effective hub genes in the pathogenesis of diabetic dyslipidemia status and highlighted potent druggable cut-point protein for designing novel therapeutic strategies based on bioinformatics analysis and molecular docking.

## Material and methods

### Participants

In this study, 80 subjects were assessed based on the eligibility criteria. In addition, among these 80 subjects, 20 individuals were excluded (Fig. 5). This study enrolled 60 women with type 2 diabetes and dyslipidemia referred to Shiraz Medical Center. Inclusion criteria included the cases diagnosed as diabetic with a minimum of 6 months of diabetes, females who agreed to participate, aged 40 to 60 years, with no history of cardiovascular disease, no blood pressure higher than 160/95 mmHg, no smoking, no supplementation. In addition, exclusion criteria included breastfeeding, hypersensitivity to propolis, pregnancy, and regular exercise program in the last 6 months. After completing the informed consent, the 60 women with type 2 diabetes and dyslipidemia were randomly distributed into four groups such as (1) the patients who did not apply the combined training and 500 mg propolis capsules supplement, which we considered the control group (Control group); (2) subjects performed combined training (EXR group); (3) subjects received the 500 mg propolis supplement capsules (SUPP group); (4) subjects performed combined training along with receiving the 500 mg propolis supplement capsules (EXR + SUPP group).

### The protocol of the blinding and randomization

The sample size was estimated at 15% alpha levels of 0.05 and 85% power. In this study, we assessed that 60 individuals (each group 15) would be required to recognize the difference between each group. In this study, the analyzer, statistician, expert assistant, trainer, and expert physiologist were blinded to the entire study process. After recruiting the cases, the statistician generated the code for each participant by creating a randomization list website (https://www.sealedenvelope.com/simple-randomiser/v1/lists). Based on our protocols, we used blocked randomization to allocate each group participant. The 60 individuals were randomly divided into the 4 and 8 block sizes.

### Study design

In this study, we performed a controlled clinical trial, single-blinding, randomized, prospective, and pre–post-intervention study to assess 8 weeks of combined training and 500 mg propolis supplement consumption in women with Diabetic dyslipidemia [36–38]. All patients who fulfilled the inclusion criteria provided written informed permission. In addition, in Table 1 we indicated the dietary intake of patients based on the dietary questionnaires completed via interview (3 days 24-h dietary recall in a week) at the pre-test and post-test. Moreover, we analyzed the closest microarray datasets obtained from the GEO database to determine effective hub genes in the pathogenesis of diabetic dyslipidemia status, specify potential markers for monitoring symptoms, and highlight potent druggable cut-point protein for designing novel therapeutic strategies. Hence, we designed a protein–protein interactions (PPIs) network based on evidence and interaction scores.

**Table 1:** Dietary intake

Factors	Control		EXR		SUPP		EXR + SUPP		*P*-value
	First week	8th week	First week	8th week		8th week	First week	8th week	
Energy (kcal)	1777.5 ± 135	1748.28 ± 143	1687.6 ± 133	1674. 36 ± 132	1692. 23 ± 149	1708. 46 ± 128	1682.89 ± 135	1712.66 ± 142	*P* > 0.05
Protein (g)	80 ± 13.42	82.65 ± 14.75	78.4 ± 13.5	80.4 ± 11.3	73.5 ± 17.2	73.5 ± 17.3	75.5 ± 12.1	79.2 ± 11.26	
Carbohydrate (g)	262.86 ± 38.48	256.32 ± 42.26	223.2 ± 40.6	242.6 ± 35.7	225.93 ± 59.2	230.82 ± 35.1	244.07 ± 52.4	240.29 ± 32.1	
Fat (g)	45.06 ± 13.2	43.6 ± 12.21	44.2 ± 14.1	42.6 ± 18.2	48.3 ± 13.2	52.26 ± 12.1	45.33 ± 12.78	48.26 ± 11.62	
Fiber (g)	14.18 ± 4.2	15.35 ± 2.54	13.2 ± 3.6	14.8 ± 3.21	13.2 ± 4.45	14.1 ± 3.56	12.75 ± 3.78	13.32 ± 2.91	

On the other hand, we predicted binding affinity numerate between effective bioactive compounds of propolis on the cut-point proteins in the PPIs network by molecular docking method. In this study, all protocols were completed in compliance with the Islamic Azad University Isfahan (Khorasgan) branch research ethics committee (IR.IAU.KHUISF.REC.1400.265). Furthermore, this study was registered in the Iranian Registry of Clinical Trials; IRCT code: IRCT20211229053561N1.

### In silico network analysis

To signify principal genes involved in type 2 diabetes (T2D) pathogenesis along with dyslipidemia and define the hub genes with significant differential expression in T2D conditions, we analyzed genes expression of microarray dataset, GSE156993, in adults with type 2 diabetes using the Bioconductor-based on Limma package, and MAS5 normalization in the R programming language. Based on the information from the dataset, we selected five samples of T2DM with dyslipidemia properties as T2D status compared with five healthy subjects. Based on adjusted *P.*value < *0.05* and logFC ± 0.5 for GSE156993 outputs, we recognized significant differential gene expression with a distinct pattern of up and down-regulation. We have shown the results of data analysis (significant differential gene expression with *P.*value < *0.001*) in the R programming language in the form of a heatmap diagram. Protein–protein interactions network construction was performed in the STRING 11.5 database based on the middle confidence score [39]. Visualized parameters of the PPI network, including degree = 10, betweenness centrality = 0.004, and closeness centrality = 0.3, were applied in the CytoScape software to highlight hub genes [40]. Among 128 hub genes, we selected ten functionally related genes based on the PPIs network, literature review, and molecular signaling pathway enrichment.

The network's parameters analysis showed ADIPOQ and IL-6 with the highest degree, and the most betweenness centrality in the grid could be druggable nodes for treating T2D with dyslipidemia [41]. Hence, we could signify the interactive and collaborative hub genes in the molecular signaling pathways involved in the T2D status based on intelligent prioritization and analytic visualization algorithms in the DAVID database [42], KOBAS-i [43], Wiki Pathway [44], Reactome [45], Panther databases [46] and Enrich-r [41].

### Virtual screening and docking methods

Based on bioinformatics analysis and enrichment tools, we manifested that ADIPOQ and IL-6 are cut-point nodes in the hub genes network and could be druggable drug design and treatment candidates. Accordingly, we browsed the UniProt database [47] and Protein Data Bank (PDB) database [48] to select the sufficiently quality three-dimensional (3D) structure of ADIPOQ (6U66) and IL-6 (1ALU). Preparation and optimization of the proteins' 3D structure consisting of the removal of extra chains, ligands, and non-complex ions were conducted in the Chimera 1.8.1 software [49]. Furthermore, we prepared a list of effective bioactive compounds derived from propolis, including naringenin, cinnamic acid, chalcone, kaempferol, chrysin, galangin, tectochrysin, and sakuranetin. The SDF format of the bioactive compounds' 3D structures was obtained from the PubChem database [50], and a bioactive compounds library was prepared using Open Bable software [51].

The molecular docking method was conducted in the PyRx software to determine suitably binding affinity between small molecules and druggable candidates in the dimensional search space. In this study, we authorized binding affinity score < − 5 and RMSD < 2 as suitable affinity [52, 53]. For the bioactive compounds of this study, we browsed the SwissADME database [54] and investigated Lipinski drug-likeness parameters.

### Consumption of propolis

The subjects who received the propolis supplementation were blinded to the individual grouping. Each dosage of the propolis supplementation contained 500 mg in the form of capsules [38]. The propolis supplementation was consumed three times a day (morning, noon, and night) after each meal for 8 weeks [38].

### Exercise training protocol

The exercise training protocol was performed in 3 sessions/week for 8 weeks. It should be noted that the supplement and placebo groups did not participate in any exercise training during the study period. The combined training protocol included 30–50 min of aerobic training (50–70% Vo2_max_) and 45 min of resistance training [36, 37]. Following the overload principle, exercises were performed for 8 weeks from low to high intensity. In the initial sessions, participants cycled for 30–50 min at 50–70% of maximal heart rate after 5 min of warm-up and gradually increased to 80% [38]. The polar heart rate monitor controlled the exercise intensity (Polar Campbell Finometer, Finland).

Moreover, resistance training consisted of eight stations (8–12 repetitions), including Barbell Curl, Lying Dumbbell Tricep Extension, Pullups, Side Lateral Raise, Barbell Bench Press, Leg Extensions, Seated Leg Curl, and Standing Calf Raise Machine [37]. The intensity of resistance training was determined based on subjects results from a one-repetition maximum (1RM). It should be noted that the exercise training protocol was executed and supervised by expert sports physiologies. Moreover, the data analyzer, statistician, and expert assistant were blinded in this study.

### Biochemical index measurement

The blood collection was conducted at the beginning and end of this trial. It should be noted that the participants were fasted for 12 h before blood taking. 10 mL of peripheral blood was collected from each patient; blood samples were submitted to a laboratory for medical diagnosis using the following safety precautions and cold chain. Blood samples were centrifuged at 1200*g* for 10 min at room temperature to extract the serum and frozen at − 70 °C. The level concentration of superoxide dismutase (SOD, Randox, SD125), malondialdehyde (MDA, Bioassay Technology Laboratory, E2186Hu), total antioxidant capacity (TAC, Randox, NX2332 UK), interleukin-6 (IL6, Bioassay Technology Laboratory, E0090Hu), secreted frizzled-related protein 5 (SFRP5, Bioassay Technology Laboratory, E2186Hu), and C1q/TNF-related Protein-12 (CTRP12, Bioassay Technology Laboratory, E4306Hu) were measured before and after the intervention using the enzyme-linked immunosorbent assay (ELISA) technique. Moreover, we measured the concentration of lipid profiles (Pars Azmon, Iran), such as cholesterol, triglycerides (TG), high-density lipoprotein (HDL), low-density lipoprotein (LDL) by enzymatic assay kits, and adiponectin (Abcam, ab99968), and insulin (Abcam, ab100578) were measured by ELISA technique.

### Statistical analysis

We estimated the sample size based on an alpha level of 0.05 and 85% power. Furthermore, based on the analysis, 60 cases (each group of 15) were required to analyze the difference between each group. Moreover, data analysis was performed by repeated measures ANOVA followed by Tukey's post hoc for between-group comparison. In addition, the intra-group was analyzed by *t*-tests using Graph Pad Prism statistical software, version 9 (Graph Pad, San Diego, CA, USA). In this study, the normalization was conducted by Kolmogorov–Smirnov. As a result, *P.*value ≤ *0.05* was demonstrated to be statistically significant and defined as the mean ± SD.

## Results

### Protein–protein network construction and social network analysis

**Fig. 1:**
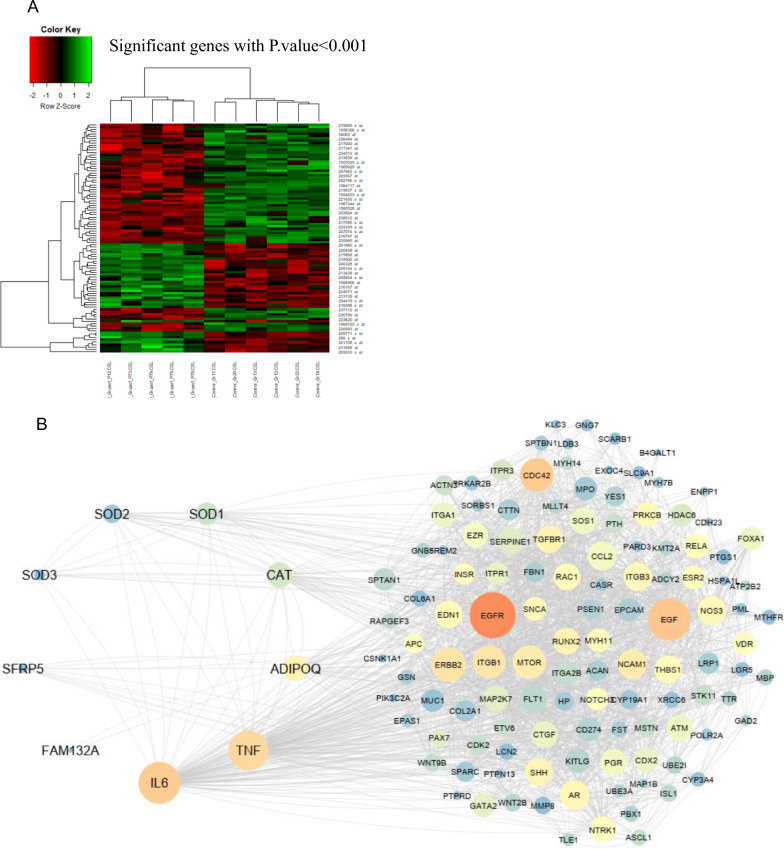
Protein–protein network construction and social network analysis. **A** The heatmap plot of significant genes with differential expression demonstrated the comparison of gene expression of healthy subjects to adult diabetic patients with dyslipidemia considering *P.*value < *0.001* thresholds. **B** Construction of the protein–protein interactions network of significant differential expressions involved in adult diabetic dyslipidemia status considering the visualizing parameters of the network. In this network, genes were ranked based on degree and betweenness centrality. The most prominent node indicated a higher degree, and the orange indicated genes with the highest betweenness centrality. IL-6 and ADIPOQ are the master regulatory cut-point genes as druggable candidates for pharmacological approaches and modulating pathogenesis networks in this network

**Fig. 2:**
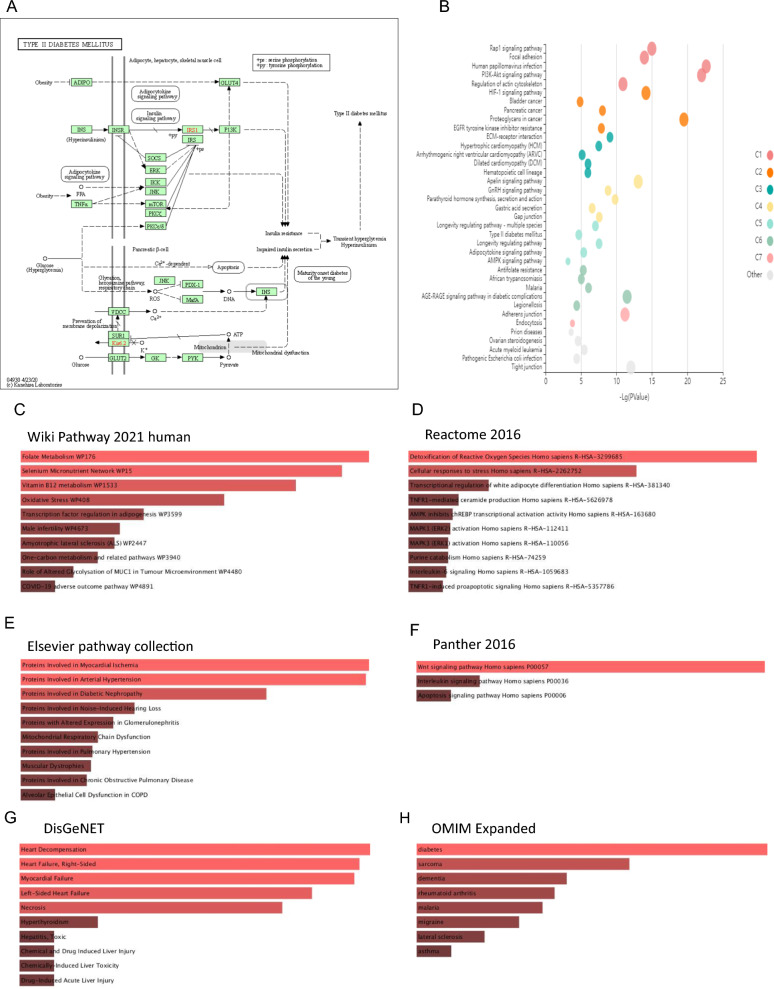
Functional and molecular signaling pathway enrichment analysis. **A** The DAVID databases analysis suggested that most hub genes are involved in the etiology of type 2 diabetes mellitus. ADIPOQ initiates this pathogenic pathway. **B**–**F** Oxidative stress, detoxification of reactive oxygen species, cellular response to stress, heart ischemia, hypertension, diabetes, Wnt signaling pathway, apoptosis, and adipocytokine signaling pathway were also highlighted by the KOBAS-i, Wiki Pathway, Reactome, Elsevier pathway collection, and Panther databases. **G**, **H** Heart dysfunction and diabetes were identified as pathogenic processes associated with FAM132A, SFRP5, ADIPOQ, TNF, IL-6, C1QTNF1, CAT, SOD1, SOD2, and SOD3 by enrichment of hub genes in the DisGeNET and OMIM databases

In this bioinformatics study, the microarray dataset analysis of adult diabetic patients with dyslipidemia showed that among 1812 genes with significant differential expression threshold *P.*value < *0.05*, 913 genes were down-regulated, and 690 genes were overexpressed (logFC ± 0.5). Significant differential expression of genes in the adult diabetic with dyslipidemia was indicated in the heatmap diagram (Fig. 1A). A network of significant genes (up/down-regulated) was designed using the STRING 11.5 database. After applying the visualize parameters of network analysis, degree = 10, betweenness centrality = 0.004, and closeness centrality = 0.3, 128 hub genes were characterized by operating CytoScape software (Fig. 1B). We selected FAM132A, SFRP5, ADIPOQ, TNF, IL-6, C1QTNF1, CAT, SOD1, SOD2, and SOD3 from 128 hub genes for the experimental validation. Hence, a PPIs network of selected genes for detecting master genes and druggable candidates was designed (Fig. 1B). Enrichment of hub genes in the enrichment tools indicated that these genes are related to the type 2 diabetes mellitus pathogenesis pathway based on the DAVID database algorithm. Also, ADIPOQ is a master gene for initiating this pathogenesis pathway (Fig. 2A). Moreover, KOBAS-i, Wiki Pathway, Reactome, Elsevier pathway collection, and Panther databases highlighted oxidative stress, detoxification of reactive oxygen species, cellular response to stress, proteins involved in heart ischemia, proteins involved in hypertension, proteins involved in diabetes, Wnt signaling pathway, apoptosis, and adipocytokine signaling pathway (Fig. 2B–F). Correspondingly, enrichment of hub genes in the DisGeNET [55] and OMIM databases specified heart dysfunction and diabetes as pathogenic mechanisms related to the FAM132A, SFRP5, ADIPOQ, TNF, IL-6, C1QTNF1, CAT, SOD1, SOD2, and SOD3 (Fig. 2G and H).

### Virtual screening of binding affinity between proteins and ligands

**Fig. 3:**
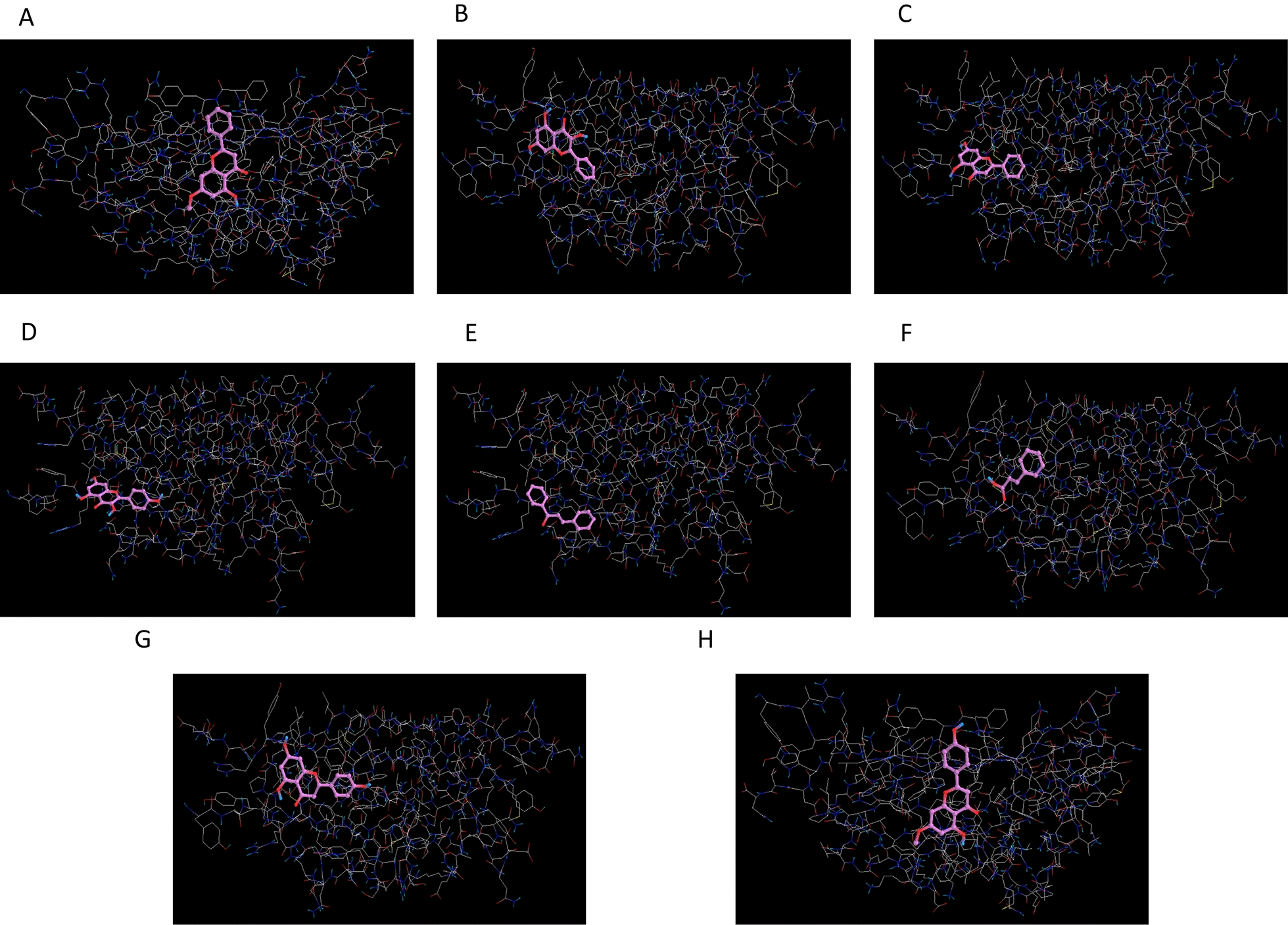
Virtual screening of binding affinity between ADIPOQ macromolecules and ligands. **A**–**H** Molecular docking affinity between ADIPOQ macromolecules and bioactive compounds derived from propolis as small molecules/ligands were estimated in the search space with dimensions *x* = 41.3689, *y* = 52.2768, and *z* = 50.3145. All bioactive compounds derived from propolis received suitable binding affinity in molecular docking. With the highest affinity score among bioactive compounds' libraries, chrysin binds to the ADIPOQ's active site

**Fig. 4:**
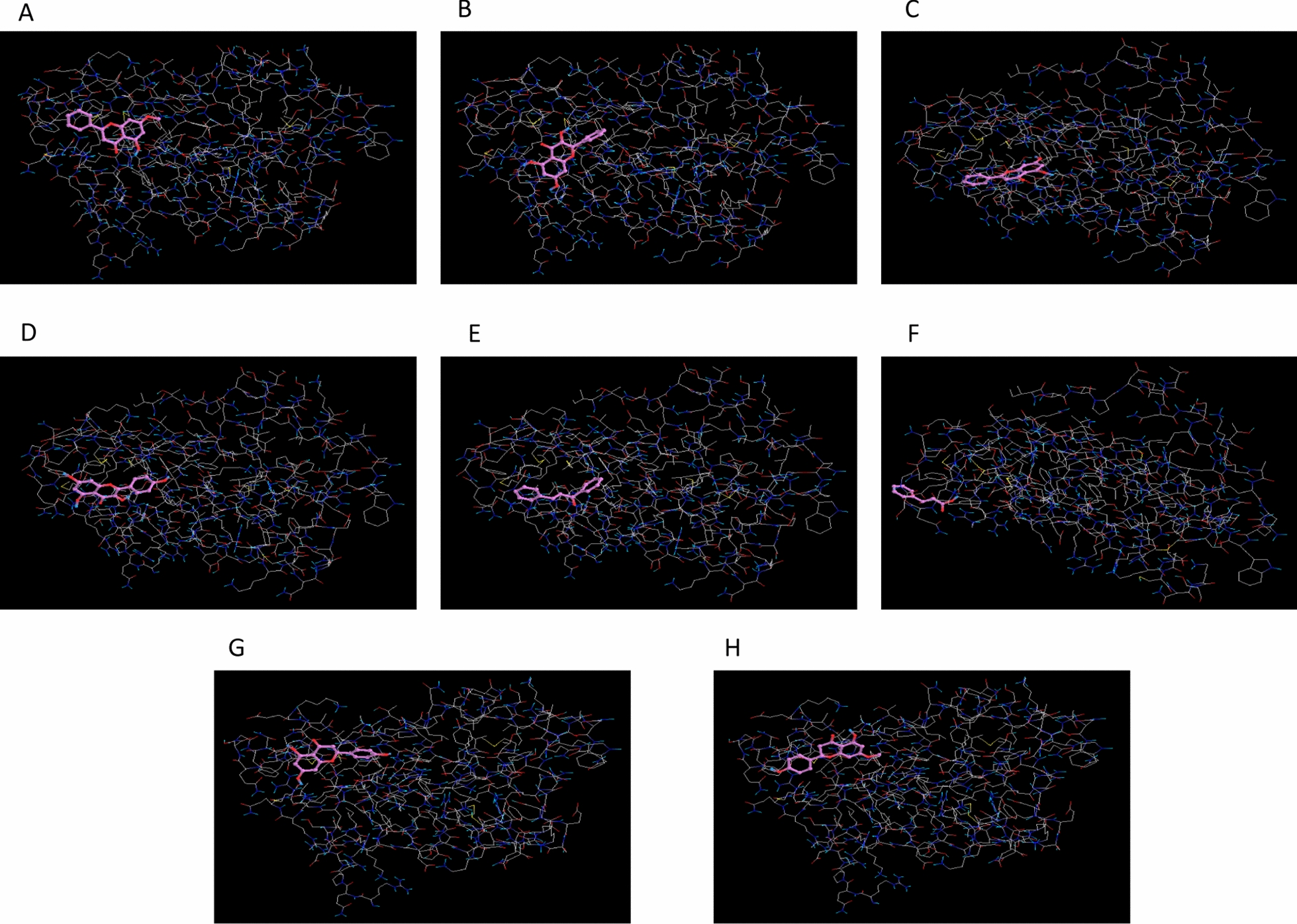
A computational chemoinformatics study to estimate the binding affinity of ligands over the IL-6 macromolecules. **A**–**H** Chemoinformatics screening in a space with dimensions (*x* = 50.1208, *y* = 43.5466, and *z* = 51.0263) evaluated the molecular docking affinity between IL-6 macromolecules and bioactive compounds obtained from propolis as small molecules/ligands. The molecular docking analysis showed that all small bioactive molecules isolated from propolis had received acceptable binding affinities scores. Kaempferol binds to the active site of IL-6 and has the library's highest affinity score of all small bioactive molecules

Based on the PPIs network analysis of hub genes, we predicted ADIPOQ and IL-6 as cut-point nodes and suitable genes/proteins for affecting the network function. According to these data, molecular docking was conducted to estimate binding affinity between ADIPOQ and IL-6 as macromolecules and bioactive compounds derived from propolis as small molecules/ligands. The output of molecular docking is presented in Fig. 3A–H for ADIPOQ, Fig. 4A–H for IL-6, and Table 2. Based on molecular docking, we predicted the most binding affinity between chrysin and ADIPOQ. Moreover, kaempferol, with the highest affinity score among bioactive compounds' libraries, binds to the IL-6's active site. It is worth noting that all bioactive compounds received suitable binding affinity in the molecular docking and could present as influential factors that modulate network function (Table 2). Based on the swissADME database analysis, chrysin, with high absorption in the gastrointestinal (GI) and permeation ability in the blood–brain barrier (BBB), has no violation of the drug-likeness parameters of Lipinski. Also, we found that kaempferol, as a soluble compound with a high GI absorption rate and no BBB permeation, has no violation and alert in Lipinski, PAINS, Brenk and leadlikeness. Hence, kaempferol as a drug-like candidate could present a practical therapeutic approach (Table 3).

**Table 2:** Molecular docking scores

PubChem ID	Bioactive compound name	Binding affinity score		RMSD
		ADIPOQ	IL-6	
5281954	Tectochrysin	− 6.3 kcal/mol	− 6.4 kcal/mol	< 2
5281616	Galangin	− 6.2 kcal/mol	− 6.2 kcal/mol	< 2
5281607	Chrysin	− 6.4 kcal/mol	− 6.4 kcal/mol	< 2
5280863	Kaempferol	− 6.1 kcal/mol	− 6.5 kcal/mol	< 2
637760	Chalcone	− 6.3 kcal/mol	− 6.2 kcal/mol	< 2
444539	Cinnamic acid	− 5.2 kcal/mol	− 5.6 kcal/mol	< 2
439246	Naringenin	− 6.1 kcal/mol	− 6.4 kcal/mol	< 2
73571	Sakuranetin	− 6.3 kcal/mol	− 6.4 kcal/mol	< 2

**Table 3:** Physicochemical properties, solubility, pharmacokinetics, drug-likeness, and medicinal chemistry properties of propolis effective bioactive compounds are predicted by the SwissADME database

No.	Bioactive compound name	SMILES	Physicochemical properties (TPSA)	Solubility	Pharmacokinetics			Druglikeness		Medicinal chemistry		
					GI	BBB	Cytochrome P45 inhibitors	Lipinski	Bioavailability Score	PAINS	Brenk	Leadlikeness
1	Tectochrysin	COC1=CC(=C2C(=C1)OC(=CC2=O)C3=CC=CC=C3)O	59.67 Å^2^	Moderately soluble	High	Yes	Yes/no	Yes; 0 violation	0.55	0 alert	0 alert	No; 1 violation: XLOGP3 > 3.5
2	Galangin	C1 = CC = C(C = C1)C2 = C(C(= O)C3 = C(C = C(C = C3O2)O)O)O	90.90 Å^2^	Moderately soluble	High	No	Yes/no	Yes; 0 violation	0.55	0 alert	0 alert	Yes
3	Chrysin	C1=CC=C(C=C1)C2=CC(=O)C3=C(C=C(C=C3O2)O)O	70.67 Å^2^	Moderately soluble	High	Yes	Yes/no	Yes; 0 violation	0.55	0 alert	0 alert	No; 1 violation: XLOGP3 > 3.5
4	Kaempferol	C1=CC(=CC=C1C2=C(C(=O)C3=C(C=C(C=C3O2)O)O)O)O	111.13 Å^2^	Soluble	High	No	Yes/no	Yes; 0 violation	0.55	0 alert	0 alert	Yes
5	Chalcone	C1=CC=C(C=C1)C=CC(=O)C2=CC=CC=C2	17.07 Å^2^	Moderately soluble	High	Yes	Yes/no	Yes; 0 violation	0.55	0 alert	1 alert: michael_acceptor_1	No; 1 violation: MW < 250

### Demographic feature and biochemical assay

**Fig. 5:**
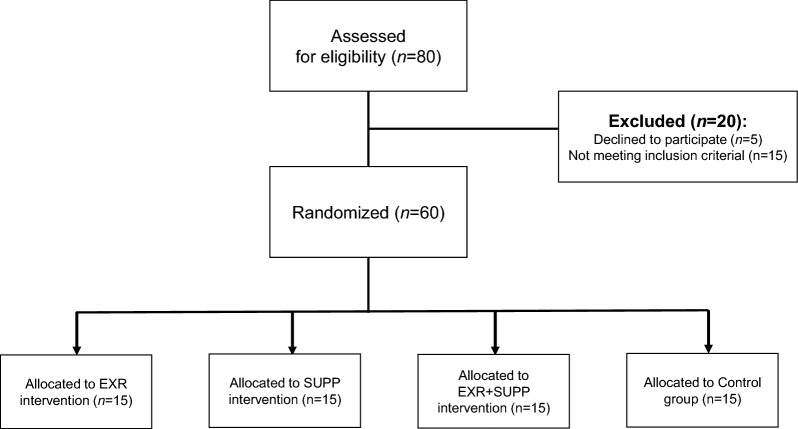
Flowchart of subject's follow-up

Women with type 2 diabetes and dyslipidemia referred to Shiraz Medical Center were recruited for this research. Based on our inclusion and exclusion criteria, 60 individuals were included in this study (Fig. 5). Moreover, the demographic data (Table 4) and Table 1 indicates no change in the age and dietary intake of each group. However, the weight and waist-to-hip ratio significantly decreased in subjects who received the 500 mg propolis supplement capsules (SUPP group), subjects who conducted combined training (EXR group), and the subjects who conducted combined training along with receiving the 500 mg propolis supplement capsules (EXR + SUPP group) compared with the Control group (Table 4, *P*.value < 0.05). In this study, we revealed that the subjects who conducted combined training along with receiving the 500 mg propolis supplement capsules (EXR + SUPP group) reduced the weight and waist-to-hip ratio vs. the other groups (Table 4, *P*.value < 0.05). In addition, we evaluated the concentration of the insulin, adiponectin, HbA1C, and lipid profiles, including cholesterol, TG, HDL, and LDL (Table 4, *P*.value < 0.05). Our data indicated that the insulin, HbA1C, and lipid profile level were diminished, and adiponectin was improved in subjects who received the 500 mg propolis supplement capsules (SUPP group), subjects who conducted combined training (EXR group), and the subjects who conducted combined training along with receiving the 500 mg propolis supplement capsules (EXR + SUPP group) compared with the Control group (Table 4, *P*.value < 0.05).

**Table 4:** The demographic and biochemical assays

	Control		*P*-value	SUPP		*P*-value	EXR		*P*-value	EXR + SUPP		*P*-value	Cutoff
	Pre-intervention	Post-intervention		Pre-intervention	Post-intervention		Pre-intervention	Post-intervention		Pre-intervention	Post-intervention		
Age	53.67	53.67	= .4	52.53 ± 3.4	52.53 ± 3.4	= .32	51.67 ± 3.4	51.67 ± 3.4	= .62	54.07 ± 3.4	54.07 ± 3.4	= .62	
Weight (kg)	77.92 ± 2.1	78.80 ± 3.1	= .18	78.42 ± 2.4	75.40 ± 1.44^*^	< 0.05	79.12 ± 4.2	74.33 ± 3.78^*^	< 0.05	77.94 ± 2.98	69.67 ± 3.52^*#^	< 0.05	
Waist-to-hip ratio (WHR)	0.97 ± 0.04	0.98 ± 0.049	= .87	0.98 ± 0.038	0.9427 ± 0.071^*^	< 0.05	0.97 ± 0.038	0.93 ± 0.046^*^	< 0.05	0.9821 ± 0.021	0.867 ± 0.039^*#^	< 0.05	≥ 0.85
HbA1C %	8.99 ± 4.38	8.29 ± 3.27	= .49	8. 9 ± 2.36	7.09 ± 1.43^*^	< 0.05	9.11 ± 3.46	7.66 ± 2.33^*^	< 0.05	8.65 ± 3.52	6.56 ± 1.2^*#^	< 0.05	6.5%
Insulin (µU/mL)	12.36 ± 1.95	12.76 ± 1.72	= .42	11.23 ± 1.12	8.34 ± 2.01^*^	< 0.05	11.53 ± 0.97	8.67 ± 0.95^*^	< 0.05	11.87 ± 1.15	6.54 ± 2.43^*#^	< 0.05	12.94 µU/mL
Adiponectin (µg/m)	2.21 ± 0.13	2.41 ± 0.23	= .51	2.81 ± 0.44	3.41 ± 0.34^*^	< 0.05	2.16 ± 0.12	3.81 ± 0.44^*^	< 0.05	2.92 ± 0.36	5.61 ± 0.31^*#^	< 0.05	6.65 µg/ml
Cholesterol (mg/dL)	250.17 ± 2.17	248.67 ± 4.45	= .19	247.46 ± 3.15	249.53 ± 2.83^*^	< 0.05	251.81 ± 1.26	242.80 ± 2.20^*^	< 0.05	251.21 ± 3.02	237.87 ± 2.87^*#^	< 0.05	≥ 200 mg/dL
HDL (mg/dL)	45.51 ± 1.19	46.47 ± 1.96	= .35	44.42 ± 2.21	51.80 ± 1.35^*^	< 0.05	46.74 ± 3.29	52.93 ± 3.16^*^	< 0.05	48.13 ± 2.24	54.87 ± 2.67^*#^	< 0.05	< 50 mg/dL
LDL (mg/dL)	141.12 ± 6.78	140.07 ± 6.87	= .43	139.15 ± 4.42	127.13 ± 3.12^*^	< 0.05	142. 7 ± 2.21	135.20 ± 2.56^*^	< 0.05	141.66 ± 5.21	120.33 ± 5.27^*#^	< 0.05	≥ 130 mg/dL
SBP/DBP (mmHg)	139.89 ± 0.56	138.89 ± 0.48	= .29	136.85 ± 0.82	129.80 ± 0.75^*^	< 0.05	142.65 ± 1.39	135.85 ± 0.56^*^	< 0.05	135.95 ± 1.29	122.80 ± 0.88^*#^	< 0.05	≥ 130/80

*Indicated the significant difference between control and other groups, *P*.value ≤ .05 was statistically significant and defined as the mean ± SD

^#^Indicated the significant difference EXR + SUPP compared with SUPP and EXR group, *P*.value ≤ .05 was statistically significant and defined as the mean ± SD

Interestingly, the subjects who performed combined training along with receiving the propolis supplement (EXR + SUPP) significantly improved the concentration of insulin, adiponectin, HbA1C, and lipid profiles compared with other groups (Table 4, *P*.value < 0.05). Notably, the subjects who performed combined training (EXR) were insignificantly compared to those who received the propolis supplement (SUPP) (Table 4, *P*.value < 0.05). However, the insulin, HbA1C, and lipid profile concentration decreased in subjects who received the 500 mg propolis supplement capsules (SUPP group) and subjects who performed combined training (EXR group) compared with the Control group (Table 4, *P*.value < 0.05). Based on these data, we could conclude that combined training along with receiving the 500 mg propolis supplement capsules (EXR + SUPP group) was more effective than the received the 500 mg propolis supplement capsules (SUPP group) and performed combined training (EXR group) (Table 4, *P*.value < 0.05).

It should be noted that, in this study, we found that the consumption of propolis did not have side effects for each patient based on simulation methods, anthropometric indexes, and biochemical parameters.

### The antioxidative system was modified by physical activity and supplementation

**Fig. 6:**
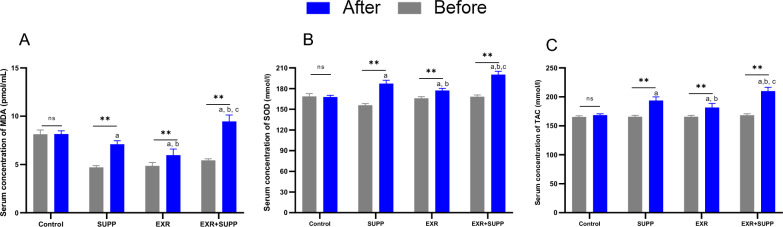
Combined training and propolis supplement altered the antioxidative markers in diabetic women with dyslipidemia. **A**–**C** The MDA, SOD, and TAC concentration in diabetic women with dyslipidemia before and after the intervention. a: indicated statistically significant difference with the Control group. b: indicated statistically significant difference with the SUPP group. c: indicated a statistically significant difference with the EXR group. *: indicated the significant difference before and after intervention in each group. a: indicated the significant difference between the supp group and the control group. b: indicated the significant difference between the Supp group and the EXR group. c: indicated the significant difference between the EXR + supp group and the Supp group and EXR group

We found that the SOD and TAC concentration was upregulated, and MDA concentration was down-regulated after receiving the propolis supplement (SUPP group) compared to before the propolis supplement (Fig. 6A–C, *P*.value < 0.05). Moreover, combined training (EXR group) enhanced SOD and TAC concentration and decreased the MDA level from baseline to the end of the intervention (Fig. 6A–C, *P*.value < 0.05). The subjects who performed combined training and received the propolis supplement (EXR + SUPP group) significantly increased SOD and TAC and reduced the MDA level compared with other groups (Fig. 6A–C, *P*.value < 0.05).

### Physical activity and supplementation improved CTRP-12, SFRP5, and the IL-6 concentration

**Fig. 7:**
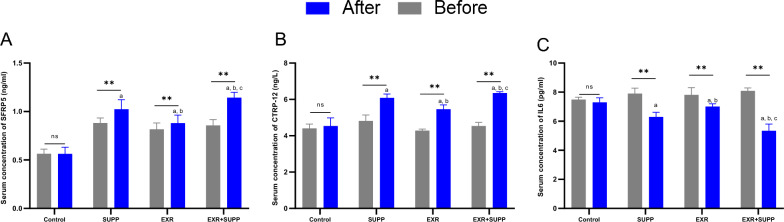
Alteration of the anti-inflammation activity and inflammation status in diabetic women with dyslipidemia. **A**–**C** The CTRP-12, SFRP5, and IL-f concentration levels in diabetic women with dyslipidemia before and after the intervention. a: indicated statistically significant differences compared with the Control group. b: indicated a statistically significant difference compared with the SUPP group. c: indicated a statistically significant difference compared with the EXR group. *: indicated the significant difference before and after intervention in each group. a: indicated the significant difference between the supp group and the control group. b: indicated the significant difference between the Supp group and the EXR group. c: indicated the significant difference between the EXR + supp group and the Supp group and EXR group

Based on our data, the serum concentrations of CTRP-12 and SFRP5 were increased after consumption of the propolis supplement (SUPP group) compared to before receiving the supplement (Fig. 7A and B, *P*.value < 0.05). Furthermore, combined training (EXR group) also increased the serum concentration of CTRP12 and SFRP5 from baseline to the end of the intervention (Fig. 7A and B, *P*.value < 0.05). Notably, subjects who performed combined training along with receiving the propolis supplement (EXR + SUPP group) enhanced the concentration of CTRP12 and SFRP5 compared with other groups (Fig. 7A and B, *P*.value < 0.05). Therefore, combined training and consumption of propolis supplements (EXR + SUPP group) might ameliorate the serum concentration of CTRP12 and SFRP5 and have anti-inflammatory activity (Fig. 7A and B, *P*.value < 0.05). Moreover, the serum concentration of IL-6 declined with consumption of the propolis supplement (SUPP group) and combined training (EXR group) compared to before the intervention (Fig. 7C, *P*.value < 0.05). I7 subjects who performed combined training along with receiving the propolis supplement (EXR + SUPP group) significantly decreased the serum concentration of IL-6 (Fig. 7C, *P*.value < 0.05). Hence, we demonstrated that anti-inflammation activity (CTRP12 and SFRP5) and inflammation (IL-6) concentration were ameliorated by combined training and receiving the propolis supplement (EXR + SUPP group).

## Discussion

In this study, we evaluated the effect of the combined exercise training protocol (aerobic and resistance training) along with consumption of the 500 mg propolis on the MDA, SOD, TAC, CTRP12, SFRP5 (as anti-inflammation agents), and IL-6 concentration in diabetic dyslipidemia status in adult women. Here, we indicated that the MDA, SOD, and TAC concentration improved when the women with type 2 diabetes and dyslipidemia performed combined training and received the propolis supplement (EXR + SUPP group). Moreover, we found that combined training along with receiving the 500 mg propolis supplement capsules (EXR + SUPP group) upregulated anti-inflammatory agents such as CTRP12 and SFRP5 and reduced the IL-6 concentration. Notably, insulin and adiponectin concentrations in women with T2D and dyslipidemia were improved by combined training and receiving the propolis supplement (EXR + SUPP group).

Analysis of the microarray dataset could create a potent insight into the differential gene expression in diseases condition compared with healthy cases. Based on this theory, we analyzed the closest microarray dataset about diabetic dyslipidemia conditions and highlighted genes with significant differential expression in pathological status compared to healthy subjects. Furthermore, analysis of significant genes based on visualizing parameters of the PPIs network prepared the essential hub genes to list with the highest degree, closeness centrality, and betweenness centrality parameters involved in the pathogenesis of diabetic dyslipidemia states.

On the other hand, based on the enrichment of hub genes, we indicated that significant hub genes are associated with oxidative stress, detoxification of reactive oxygen species, cellular response to stress, proteins involved in heart ischemia, proteins involved in hypertension, proteins involved in diabetes, Wnt signaling pathway, apoptosis, and adipocytokine signaling pathway. According to the enrichment results, it could be suggested that CTRP12, SFRP5, ADIPOQ, TNF, IL-6, C1QTNF1, CAT, SOD1, SOD2, and SOD3 hub genes and molecular signaling pathways play a pivotal role involved in diabetic dyslipidemia pathogenesis. Ouchi et al. 2010 reported that SFRP5, a novel adipokine with anti-inflammatory activity, is overly expressed in white adipose tissue and distributed in the plasma [56]. Moreover, they noted that after 24 weeks of nourishing a high-fat/high-sucrose diet for wild-type mice, leptin deficiency mice (ob/ob) and Zucker diabetic fatty mice (fa/fa) resulted in obesity phenotype, down-regulation of SFRP5 expression, and overexpression of WNT5A [56]. These data proposed that regulation of SFRP5 expression is associated with obesity states.

Furthermore, Ouchi et al. also found that SFRP5-deficient (SFRP5/^−^) mice fed a high-fat diet had even more fatty tissue inflammation triggered by macrophages and insulin resistance than wild-type mice. Adipose tissue inflammation triggers macrophage activation and insulin resistance because of the over-activation of the WNT5A [56]. Hu ZP et al. revealed a negative correlation between SFRP5 plasma concentrations and body fat variables such as BMI, waist circumference, and waist-to-hip ratio in Chinese volunteer obese individuals compared to normal-weight people. In addition, there was a negative relevance between the SFRP5 plasma concentration and insulin resistance in individuals with T2D, and SFRP5 had a protective effect that might act against the mechanism of T2DM pathogenesis [57].

On the other hand, CTRP12 was identified as an anti-inflammatory with insulin-sensitizing properties that could improve glucose metabolism. Furthermore, in obese mice whose fed diet caused weight gain, adipolin administered systemically reduced glucose intolerance and insulin resistance [26]. These findings show that anti-inflammatory adipokine, adipolin, positively affects glucose metabolism [17]. Based on this evidence, Takashi Enomoto et al. suggested that adipolin represented a novel targetable molecule for treating insulin resistance and diabetes [58].

A literature review on diets and supplements revealed various natural and chemical factors in preventing and treating diabetic dyslipidemia. For example, Mansouri et al. reported that 6.9% of college students had hypertension and a statistically significant inverse association between the risk of hypertension and dairy intake [59].

Our new results indicated that propolis could bind to ADIPOQ cut-point protein with the highest binding affinity score based on molecular docking prediction. Furthermore, based on the virtual screening of effective compounds by molecular docking method, we found that chrysin, among bioactive compounds' libraries of propolis, binds to the ADIPOQ's active site with the highest affinity score. Hence, we suggest this single ligand might be an effective natural complementary medicine for progressive metabolic disorders' prevention, management, and therapeutic strategy. Further, one of the natural pharmacological bioactive compounds identified in propolis is sakuranetin. Monika Stompor, in her review report, exhibited that sakuranetin has various functions in biological processes, such as anti-tumor activity via angiogenesis modulation, inducing apoptosis, and control of cell proliferation [60]. In addition, sakuranetin's antioxidative stress and anti-inflammatory properties are associated with reduced TNFα, IL1β, and MCP1 levels and inhibited ERK, STAT3, JNK, and p38 signaling pathways [61].

Moreover, a randomized controlled clinical trial by Pahlavani et al*.* studied the effect of simultaneous administration of propolis and 1000 mg/dl (high dose) of melatonin on inflammation, oxidative stress features, and clinical terms of primary sepsis. Faster recovery, shorter hospital stay duration, and lower death rates were reported for the pre-infectious status of sepsis ICU patients who received propolis + a high melatonin dosage (1000 mg/dl) [62]. In addition, TNF-α, IL-6, and IL-1β levels have been revealed to return to normal levels by propolis administration, and infections in diabetic foot ulcers have been proven to improve [63, 64].

Further, Kolahdouz-Mohammadi and coworkers examined the effect of egg intake on blood pressure in 748 adult participants. Meta-analysis output pinpointed that egg intake does not significantly affect adult individuals' systolic and diastolic blood pressure [65].

On the other hand, the prevalence of sitting time and a sedentary lifestyle has increased recently [1]. Human activity has diminished due to advanced technology and modern civilization, and severe ailments such as T2D, cardiovascular diseases, obesity, metabolic (dysfunction)-associated fatty liver disease (MAFLD), depression, and metabolic syndrome have increased [66–68]. In addition, accumulating adipose tissue and dyslipidemia can increase free radicals, peroxidation of fatty acids, and cytokine storms [1]. Exercise (aerobic or resistance) and physical activity (PA) might prevent the progression of type 2 diabetes, obesity, and dyslipidemia [32, 69]. Over the past two decades, several studies have demonstrated that aerobic and resistance training can enhance the body's operation and improve the organs' mechanisms [31, 70, 71]. Way et al*.* found that high-intensity interval training (80–100 Vo_2max_) improved measures of central artery stiffness and 24 h blood pressure in adults more than moderate-intensity continuous training [72]. Their results show that HIIT may be preferable to conventional aerobic exercise for managing hypertension and its associated risks, even if there is a general paucity of data comparing high-intensity interval training with moderate-intensity continuous training on these outcomes, notably 24 h blood pressure [72]. Our data indicated that combined training improved the MDA, SOD, and TAC status compared to the control group. In addition, we found that the anti-inflammatory effects, such as CTRP12 and SFRP5, were significantly increased by combined training compared to baseline. Data demonstrated that the concentration of IL-6 was reduced by combined training compared to baseline and control groups. Hence, based on these data, combined training ameliorated the antioxidant status and silenced the inflammation pathomechanism.

Wei and colleagues revealed that up-regulation of the CTRP12 could modulate glucose metabolism and ameliorate insulin sensitivity [26]. Moreover, CTRP12 might halt gluconeogenesis, increase adipocyte glucose uptake, and induce the PI3K/Akt signaling pathway [26]. Evidence has indicated that exercise and physical activity with moderate–high intensity endurance for 8 weeks (5 days/week) on the treadmill might promote the Nrf2-Keap1 axis and regulate the oxidative stress pathway in the male C57BL/6 diabetic mice induced by advanced glycation end-products-rich diet and restriction of activity [31]. Furthermore, exercise could regulate reactive oxygen species (ROS) and the mitochondrial markers' overexpression [31, 32, 73]. Maryam Haghparast Azad et al*.* indicated that exercise with 70% V_O2 max_ for 8 weeks (5 sessions/week) could modulate the expression level of Cpt2, Tfam, Ppargc1α, mt-Co1, mt-Nd1, mt-Nd5, Ndufa2, and mt-Co2 in the muscle of diabetic old male C57BL/6 mice [32].

Recently, an active biological marker for type 2 diabetes and dyslipidemia has been the most crucial and significant aspect [29]. In addition, blood biochemical detection kits are accessible, exact, and simple since they are accurate, non-invasive, and more helpful and valuable than clinical symptoms in serum or plasma [29]. Therefore, CTRP12, SFRP5, and IL-6 could be biomarkers for monitoring inflammation, type 2 diabetes, and dyslipidemia. Moreover, we found that the concentration CTRP-12 and SFRP5, MDA, SOD, and TAC was improved by combined training and consumption of propolis. In addition, ADIPOQ and IL-6 could consider cut-point nodes and suitable proteins in the pathomechanism of T2D and dyslipidemia.

This study had some limitations, such as single-center and low sample size. In addition, we did not assess the relative expression profile of the hub genes. Moreover, we did not evaluate the consumption of propolis and exercise training in both males and females to determine the sex-dependent pathophysiological features. Furthermore, we recommend that the researcher may evaluate the relative expression profile according to the in silico analysis.

## Conclusion

Based on bioinformatics, chemoinformatics, and molecular assessment, we suggest effective compounds of propolis and recommended exercise protocol might be an effective natural complementary medicine for progressive metabolic disorders' prevention, management, and therapeutic strategies such as diabetes, dyslipidemia, cardiovascular diseases, and other metabolic disorders complications. On the other hand, we provided a druggable protein, ADIPOQ, for drug discovery and drug design for diabetic cures with dyslipidemia that might be interesting for pharmaceutical insight and drug design-based natural products.

## Data Availability

All raw data and materials in the Islamic Azad University Isfahan (Khorasgan) branch are available upon request.

## Abbreviations

LDL-C: Low-density lipoprotein cholesterol
HDL-C: High-density lipoprotein cholesterol
T2D: Type 2 diabetes
PPIs: Protein–protein interactions
SFRP5: Secreted frizzled-related protein 5
WNT5A: Wingless-type family member 5A
IR: Insulin resistance
IL-6: Interleukin 6
HFD: High-fat diet
AGE: Advanced glycation end-products
